# Hydroxytyrosol Alleviates Intestinal Oxidative Stress by Regulating Bile Acid Metabolism in a Piglet Model

**DOI:** 10.3390/ijms25115590

**Published:** 2024-05-21

**Authors:** Xiaobin Wen, Fan Wan, Ruqing Zhong, Liang Chen, Hongfu Zhang

**Affiliations:** State Key Laboratory of Animal Nutrition and Feeding, Institute of Animal Sciences, Chinese Academy of Agricultural Sciences, Beijing 100193, China; wenxiaobin001@126.com (X.W.); wanfanfw@126.com (F.W.); zhanghongfu@caas.cn (H.Z.)

**Keywords:** hydroxytyrosol, gut microbiota, bile acid, intestinal health, oxidative stress

## Abstract

Infants and young animals often suffer from intestinal damage caused by oxidative stress, which may adversely affect their overall health. Hydroxytyrosol, a plant polyphenol, has shown potential in decreasing intestinal oxidative stress, but its application and mechanism of action in infants and young animals are still inadequately documented. This study selected piglets as a model to investigate the alleviating effects of hydroxytyrosol on intestinal oxidative stress induced by diquat and its potential mechanism. Hydroxytyrosol improved intestinal morphology, characterized by higher villus height and villus height/crypt depth. Meanwhile, hydroxytyrosol led to higher expression of Occludin, MUC2, Nrf2, and its downstream genes, and lower expression of cytokines IL-1β, IL-6, and TNF-α. Both oxidative stress and hydroxytyrosol resulted in a higher abundance of *Clostridium_sensu_stricto_1*, and a lower abundance of *Lactobacillus* and *Streptococcus*, without a significant effect on short-chain fatty acids levels. Oxidative stress also led to disorders in bile acid (BA) metabolism, such as the lower levels of primary BAs, hyocholic acid, hyodeoxycholic acid, and tauroursodeoxycholic acid, which were partially restored by hydroxytyrosol. Correlation analysis revealed a positive correlation between these BA levels and the expression of Nrf2 and its downstream genes. Collectively, hydroxytyrosol may reduce oxidative stress-induced intestinal damage by regulating BA metabolism.

## 1. Introduction

Oxidative stress is a common physiological phenomenon that occurs in response to various stimuli, leading to excessive production of reactive oxygen species (ROS) and an imbalance in the antioxidant defense system, which can further result in cell and tissue damage [1]. Excessive ROS can damage cellular lipids, proteins, or DNA, inhibiting their normal functions, hence it is crucial to protect cells from the impacts of oxidative stress [2]. Globally, oxidative stress-related intestinal diseases are highly prevalent, particularly among infants and young animals, such as sepsis and enteritis [3,4]. The incidence of diseases associated with oxidative stress and inflammation is continuously increasing in developed countries [5]. This phenomenon is associated with various factors, including changes in lifestyle, environmental pollution, chronic stress, and dietary habits. The intestine not only serves as a site for nutrient absorption but also represents one of the most crucial protective barriers [6]. The integrity of intestinal structure and barrier function is vital to human and animal health [7]. However, due to the underdeveloped intestinal tract in infants and young animals, they are more susceptible to external factors that trigger oxidative stress, which may lead to intestinal damage [8,9,10]. Then it will exacerbate oxidative stress and inflammation, forming a vicious cycle [11]. Dietary intervention plays a crucial role in mitigating intestinal damage and promoting intestinal health [12]. Therefore, researching how to alleviate intestinal oxidative stress through dietary intervention is critically important.

Gut microbiota and their metabolites, such as short-chain fatty acids (SCFAs) and some bile acids (BAs), have been proven to play a vital role in regulating intestinal health [13]. SCFAs, such as acetate, butyrate, and propionate, are produced by gut microbiota through the metabolism of carbohydrates [14]. These SCFAs possess remarkable chemical properties and their impact on health has been extensively documented [12,15,16]. These compounds are known to modulate gut function and numerous metabolic pathways within the liver, adipose tissue, muscle, and brain [17,18,19,20]. Notably, butyrate is the primary metabolite of gut microbiota and is essential for the nutrition and protection of the mammalian gut [21]. BAs are produced from cholesterol metabolism. They are converted to primary BAs in the liver and then enter the intestine, where they are metabolized by gut microbiota into secondary BAs, maintaining homeostasis through the enterohepatic circulation [22]. BAs, especially tauroursodeoxycholic acid (TUDCA), are converted from microbial-metabolized secondary bile acids. It was found that TUDCA can activate the Nrf2 signaling pathway, upregulate the expression of antioxidant enzymes, and exert antioxidant effects, which have been validated in various disease models, such as metabolic diseases and renal ischemia-reperfusion [22,23]. Therefore, modulating gut microbiota or their metabolites (SCFAs and BAs) may represent a potential strategy for improving intestinal oxidative stress. Recent findings have suggested that polyphenolic compounds positively affect the stability of gut microbiota and their metabolic products [12,24].

Hydroxytyrosol (3,4-dihydroxybenzene ethyl alcohol) is a natural polyphenolic compound primarily found in olive fruits, leaves, and olive oil [25]. It possesses strong antioxidant capacity, with antioxidant activity 15 times that of green tea and 3 times that of coenzyme Q [26]. Previous in vitro studies have shown that hydroxytyrosol can activate the Nrf2 signaling pathway, thereby promoting the expression of various downstream antioxidant enzymes such as catalase (CAT), superoxide dismutase (SOD), NAD(P)H: quinone oxidoreductase 1 (NQO1), and heme oxygenase 1 (HO-1), aiding in resisting oxidative stress [27,28,29,30,31]. Moreover, our previous findings have demonstrated that hydroxytyrosol can modulate the gut microbiota of mice to activate the nuclear factor erythroid 2-related factor 2 (Nrf2) signaling pathway and counteract oxidative stress [32,33]. Furthermore, the Mediterranean diet deemed the healthiest due to its low prevalence of cardiovascular, aging-related, and intestinal diseases, is abundant in olive oil, a significant source of hydroxytyrosol [34,35]. Based on the above findings, hydroxytyrosol has promising potential in alleviating oxidative stress and maintaining intestinal health in infants and young animals. However, research on the application of hydroxytyrosol in infants and young animals is rare. Furthermore, a recent article reviewed the interaction between polyphenols and gut microbiota [12]. The authors summarized those polyphenols impact metabolites such as SCFAs, BAs, and tryptophan metabolism by altering gut microbiota composition, microbial enzyme activity, and other potential mechanisms. These changes in microbial metabolites induced by polyphenols subsequently play various roles in maintaining the intestinal barrier, central nervous system, and pulmonary function homeostasis. Therefore, we speculate that, as a polyphenol, hydroxytyrosol may similarly modulate gut microbiota and their metabolites to improve intestinal health. Nevertheless, research on the impact of hydroxytyrosol on gut SCFAs and BAs metabolism remains limited.

In summary, we hypothesize that hydroxytyrosol may alleviate oxidative stress and improve intestinal health by regulating the gut microbiota or their metabolic products. Given the physiological and functional similarities between pigs and humans, pigs represent an excellent model for studying the potential mechanisms of intestinal oxidative stress and therapeutic strategies [36]. Therefore, this study selected piglets as the model to investigate the alleviating effects and potential mechanisms of hydroxytyrosol on intestinal oxidative stress. The findings may provide new regulatory targets and theoretical foundations for the treatment or prevention of intestinal oxidative stress in infants and young animals.

## 2. Results

### 2.1. Hydroxytyrosol Improves Intestinal Barrier Function

**Figure 1:**
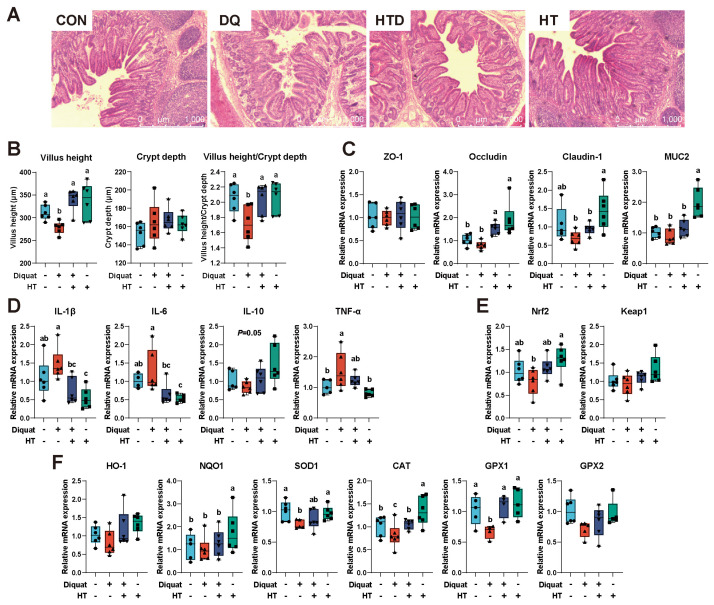
Intestinal morphology and barrier function. (**A**) Representative pictures by hematoxylin–eosin (HE) staining of ileum sections (scale bars: 1000 µm). (**B**) Statistic data of ileal villus height, crypt depth, and villus height/crypt depth. The mRNA expression of tight junction proteins (**C**), cytokine (**D**), Nrf2 (**E**), and its downstream genes (**F**) in the ileum. CON group: pigs receiving a basal diet and injected normal saline; DQ group: pigs receiving a basal diet and injected diquat (DQ; 8 mg/kg body weight); HTD group: pigs receiving a basal diet supplemented with 500 mg/kg hydroxytyrosol (HT) and injected DQ; HT group: pigs receiving a basal diet supplemented with 500 mg/kg HT and injected normal saline. IL-, interleukin-. TNF-, tumor necrosis factor-. Nrf2, nuclear factor erythroid 2-related factor 2. HO-1, heme oxygenase 1. NQO1, NAD(P)H:quinone oxidoreductase 1. SOD1, superoxide dismutase 1. CAT, catalase. GPX, glutathione peroxidase. Different colors represent different treatments, while different shapes represent different individuals. Different letters represent significant differences (*p* < 0.05).

Compared to the control group (CON), diquat (DQ) treatment resulted in damaged ileum morphology, characterized by irregular ileal structure, disordered villi arrangement, shortening, and shedding (Figure 1A). Additionally, compared to the CON group, the villus height and villus height/crypt depth in the DQ group were lower (Figure 1B, *p* < 0.05). Compared to the DQ group, hydroxytyrosol ameliorated these damages, and the villus height and villus height/crypt depth in the HT+DQ group (HTD) were higher (*p* < 0.05). Furthermore, compared to the CON group, DQ treatment did not significantly affect the expression of tight junction proteins and MUC2 (*p* > 0.05). Compared to the CON group, the HTD group exhibited higher levels of Occludin expression (*p* < 0.05). Moreover, the expressions of Occludin and MUC2 were higher when hydroxytyrosol was added alone (*p* < 0.05, Figure 1C).

### 2.2. Hydroxytyrosol Attenuates Intestinal Inflammation and Enhances Antioxidant Capacity

Compared to the CON group, DQ treatment led to higher expression of inflammatory cytokines TNF-α (*p* < 0.05). Compared to DQ treatment, hydroxytyrosol treatment resulted in lower expression of IL-1β and IL-6 (*p* < 0.05). Furthermore, compared to the CON group, hydroxytyrosol alone showed lower expression of IL-1β and IL-6 (*p* < 0.05, Figure 1D). The expression changes in Nrf2 and its downstream genes are shown in Figure 1E,F. The expressions of SOD1, CAT, and GPX1 in the DQ group were lower compared to the CON group (*p* < 0.05). While hydroxytyrosol treatment showed higher expression of CAT (*p* < 0.05) and GPX1 (*p* < 0.05) compared to the DQ group. Additionally, when compared to the control group, hydroxytyrosol alone also resulted in higher NQO1 and CAT (*p* < 0.05).

### 2.3. Hydroxytyrosol Regulates Microbial Composition

**Figure 2:**
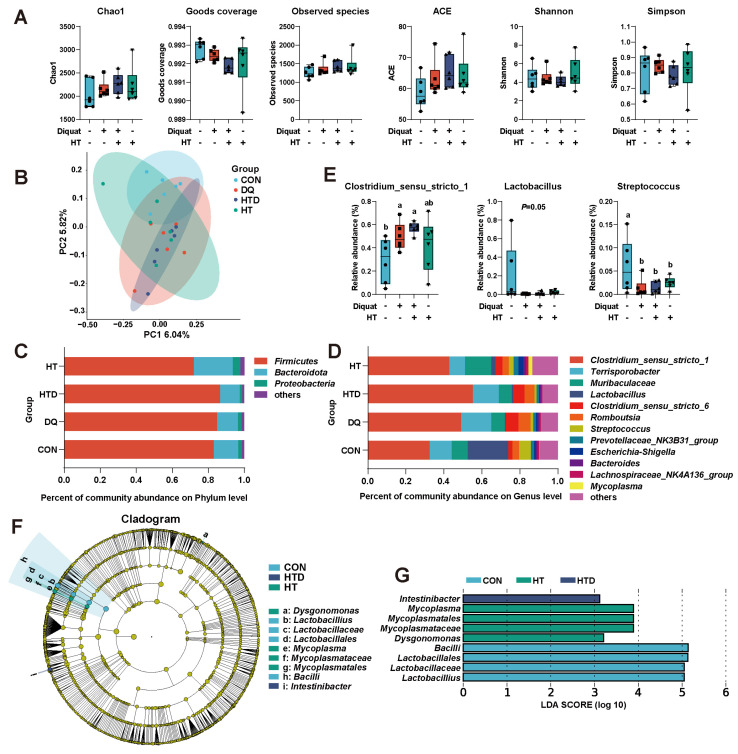
Intestinal microbial diversity and composition. (**A**) The alpha diversity indices of ileal microbiota. (**B**) PCoA plot based on binary Jaccard distances at the OTU level. Microbial composition at the (**C**) phylum and (**D**) genus level, respectively. (**E**) The relative abundance changes of differential bacteria. (**F**) Cladogram and (**G**) LDA distribution. CON group: pigs receiving a basal diet and injected normal saline; DQ group: pigs receiving a basal diet and injected diquat (DQ; 8 mg/kg body weight); HTD group: pigs receiving a basal diet supplemented with 500 mg/kg hydroxytyrosol (HT) and injected DQ; HT group: pigs receiving a basal diet supplemented with 500 mg/kg HT and injected normal saline. Different colors represent different treatments, while different shapes represent different individuals. Different letters represent significant differences (*p* < 0.05).

The alpha and beta diversities demonstrated that neither DQ stimulation nor hydroxytyrosol treatment significantly impacted microbial diversity (Figure 2A,B). At the phylum level, *Firmicutes*, *Bacteroidota*, and *Proteobacteria* collectively comprised approximately 98.39% of the total ileal bacterial community (Figure 2C). At the genus level, *Clostridium_sensu_stricto_1*, *Terrisporobacter*, *Muribaculaceae*, *Lactobacillus*, *Clostridium*_*sensu*_*stricto_6*, *Romboutsia,* and *Streptococcus* emerged as the predominant genera (Figure 2D). The results showed that at the genus level, both DQ and hydroxytyrosol treatments showed a higher relative abundance of *Clostridium_sensu_stricto_1* (*p* < 0.05), and a lower relative abundance of *Lactobacillus* (*p* = 0.05) and *Streptococcus* (*p* < 0.05, Figure 2E). Additionally, analysis using the LEfSe method to assess marker bacteria in each group revealed an enrichment of *Lactobacillus* in the CON group, *Intestinibacter* in the HTD group, and *Mycoplasma* in the HT group (Figure 2F,G).

### 2.4. Hydroxytyrosol Changes BAs Metabolism

**Figure 3:**
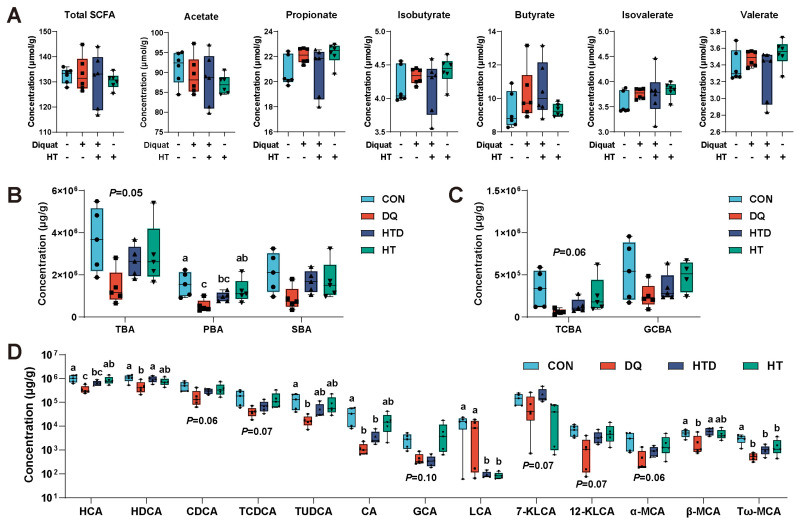
Contents of intestinal SCFAs and BAs. (**A**) Contents of SCFA in the ileum. (**B**,**C**). The alteration in each type of BA content. (**D**) The content of differential BAs. CON group: pigs receiving a basal diet and injected normal saline; DQ group: pigs receiving a basal diet and injected diquat (DQ; 8 mg/kg body weight); HTD group: pigs receiving a basal diet supplemented with 500 mg/kg hydroxytyrosol (HT) and injected DQ; HT group: pigs receiving a basal diet supplemented with 500 mg/kg HT and injected normal saline. Different colors represent different treatments, while different shapes represent different individuals. Different letters represent significant differences (*p* < 0.05).

There was no significant difference in SCFAs and total SCFAs among the groups (*p* > 0.05, Figure 3A). As can be seen from Figure 3B,C, the concentration of total bile acid (TBA), primary bile (PBA), and tauro-conjugated bile acid (TCBA) in the DQ group decreased, but they were partially recovered by hydroxytyrosol treatment. Specifically, DQ treatment resulted in lower concentrations of hyocholic acid (HCA), hyodeoxycholic acid (HDCA), tauroursodeoxycholic acid (TUDCA), cholic acid (CA), β-Muricholic acid (β-MCA) and tauro-ω-muricholic acid (Tω-MCA) (*p* < 0.05, Figure 3D). After treatment with hydroxytyrosol, the above BAs were all increased in varying degrees, with a tendency to converge to the CON group, especially HCA, HDCA, and TUDCA. In addition, chenodeoxycholic acid (CDCA), tauro-CDCA (TCDCA), 7-Ketolithocholic acid (7-KLCA), 12-KLCA and α-MCA also showed the same trend, although there was no significant difference. In addition, hydroxytyrosol treatment showed lower content of lithocholic acid (LCA) and Tω-MCA (*p* < 0.05, Figure 3D).

### 2.5. Correlation Analysis between Differential Ileal Microbiota, BAs and Gene Expression

**Figure 4:**
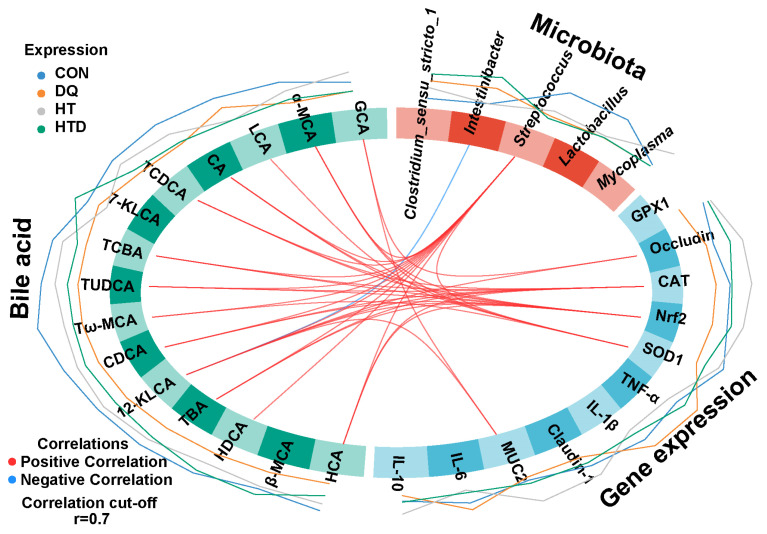
Circle plot displaying correlations between the microbiota, BAs, and gene expression. Positive and negative correlations (r > 0.7) were displayed by red and blue links, respectively. These lines demonstrate the overall expression levels of the selected variables, with lines closer to the circle indicating lower numerical values of expression. CON group: pigs receiving a basal diet and injected normal saline; DQ group: pigs receiving a basal diet and injected diquat (DQ; 8 mg/kg body weight); HTD group: pigs receiving a basal diet supplemented with 500 mg/kg hydroxytyrosol (HT) and injected DQ; HT group: pigs receiving a basal diet supplemented with 500 mg/kg HT and injected normal saline.

To further understand the relationship among microbiota, differential BAs, and gene expression, DIABLO was employed to investigate their correlations (Figure 4). *Streptococcus* was positively correlated with almost all BAs except glycocholic acid (GCA), LCA,7-KLCA, and β-MCA. Additionally, HCA and TUDCA were positively correlated with CAT and Nrf2. Furthermore, CA was positively correlated with Nrf2 and SOD1, while LCA and Tω-MCA were positively correlated with SOD1.

## 3. Discussion

Diquat (DQ), a bipyridyl herbicide, is commonly used in model systems as an inducer of in vivo oxidative stress models [37]. Its primary mechanism of action involves catalyzing the conversion of molecular oxygen into free oxygen radicals, thereby inducing oxidative stress within cells. Intestinal health is considered a crucial foundation of overall organismal health, with intestinal morphology and the expression of tight junction proteins serving as important indicators of intestinal health [38]. Previous studies have demonstrated that DQ-induced oxidative stress caused damage to intestinal morphology and compromised intestinal barrier function [39,40]. This study similarly found that DQ treatment led to the occurrence of oxidative stress, resulting in intestinal structural damage and lower villus height and villus height/crypt depth. However, hydroxytyrosol was shown to ameliorate these changes. Intestinal villus height, crypt depth, and villus height/crypt depth in the small intestine are important morphological indicators reflecting intestinal permeability, absorptive capacity, and the integrity of the intestinal mucosa [41]. These phenotypic changes align with previous research on oxidative stress-induced intestinal injury [39]. According to the results of this study, hydroxytyrosol has shown potential as an effective strategy for maintaining intestinal morphology and ameliorating the damage caused by oxidative stress. Specifically, the intestinal barrier serves as the first line of defense, effectively reducing the risk of pathogen invasion and intestinal diseases. Tight junction proteins, as critical components of the intestinal barrier, are essential for maintaining its integrity. Disruption or dysfunction of tight junction proteins in epithelial cells leads to an increase in intercellular gaps, resulting in enhanced intestinal permeability, a phenomenon commonly described as “intestinal leakage” or “leaky gut syndrome” [42]. This study found that hydroxytyrosol treatment showed higher expression of Occludin. This finding is consistent with other research results, indicating that hydroxytyrosol can increase tight junction proteins and enhance intestinal barrier function [43]. Goblet cells are not only an important component of intestinal epithelial cells that maintain intestinal epithelium integrity but also the main cells that secrete mucins. MUC2 is a key regulatory gene of the intestinal mucous layer, essential for maintaining intestinal barrier integrity. The results showed that hydroxytyrosol led to higher expression of MUC2, which indirectly reflected the acceleration of intestinal epithelial cell proliferation and the promotion of intestinal barrier regeneration [43]. In a word, HT can improve intestinal morphology and ensure the integrity of intestinal barrier function.

Oxidative stress not only triggers inflammatory responses, but the sustained presence of inflammation can also exacerbate oxidative stress, forming a vicious cycle that causes further damage to cells and tissues [44,45]. Cytokines such as tumor necrosis factor (TNF-α), interleukins (IL-1β, IL-6), and others play crucial roles in the inflammatory response by promoting cellular damage and the inflammatory process, thereby exacerbating oxidative stress. Therefore, monitoring the effects of drugs on the expression of cellular inflammatory cytokines is a key indicator for assessing the efficacy of drugs in alleviating oxidative stress. Consistent with previous findings, our study observed a higher expression of cytokines (TNF-α) under oxidative stress conditions, likely due to cellular damage caused by oxidative stress, which in turn activates the inflammatory signaling pathways. Previous research has confirmed that oxidative stress leads to increased expression of NF-κB and IRF pathways, all of which are closely associated with the inflammatory response [46,47]. In our study, we found that hydroxytyrosol decreased the expression of these cytokines, indicating its anti-inflammatory properties. Additionally, hydroxytyrosol has been shown to lower levels of IL-1β, IL-6, and TNF-α and increase the level of the anti-inflammatory cytokine IL-10 to ameliorate intestinal inflammation in mice colitis models [43]. These findings indicate that hydroxytyrosol possesses certain anti-inflammatory properties, which contribute to alleviating oxidative stress. Furthermore, cells respond to oxidative stress by activating the antioxidant signaling pathway, particularly the Nrf2 pathway, which induces the expression of antioxidant genes such as SOD, CAT, and GPX [48]. These antioxidant genes help clear reactive oxygen species and other oxidants, protecting cells from oxidative damage [49]. Therefore, monitoring the effects of drugs on the expression of antioxidant genes is also a crucial indicator for evaluating the efficacy of drugs in alleviating oxidative stress. Our previous research demonstrated that after injecting DQ into mice, the expression of Nrf2, NQO1, CAT, and GPX2 was lower, but treatment with hydroxytyrosol prevented these decreases [32]. In this study, we found that hydroxytyrosol could restore the decreased expression of Nrf2, SOD1, CAT, and GPX1 induced by DQ treatment. Additionally, it has been found in research on other organ diseases that hydroxytyrosol can activate the Nrf2 signaling pathway to enhance antioxidant capacity, such as cardiovascular diseases [30], wound healing [50], and Alzheimer’s disease [51]. In summary, hydroxytyrosol could simultaneously reduce the expression of inflammatory cytokines and enhance the expression of antioxidant genes, indicating its strong potential in alleviating oxidative stress, and may have a beneficial role in treating diseases related to oxidative stress.

The gut microbiota serves as a crucial bridge between diet and host intestinal health. Our previous study and other researchers have suggested that hydroxytyrosol may improve oxidative stress by modulating the gut microbiota [32,33,43]. However, in our study, we did not observe an improvement in the restoration of the piglet gut microbiota with hydroxytyrosol treatment. We speculate that this discrepancy may be attributed to species differences. Studies by others have been conducted in mice models, while our research was performed in piglets. To our knowledge, this is the first report to investigate the effects of hydroxytyrosol on the gut microbiota of piglets. Our speculation is plausible because there are ubiquitous physiological, metabolic pathways, and gut microbiota community differences between mice and piglets. These differences may lead to differential effects of the same compound in different species [52,53]. Additionally, individual variations, environmental factors, and dietary habits may also influence the results. In our study, hydroxytyrosol was added to piglet feed as an additive, rather than administered via aqueous solution gavage [32,33,43], which may account for the discrepancies between our findings and previous studies. Therefore, our study provides preliminary insights into the effects of hydroxytyrosol on gut microbiota in a piglet model. Future research could further explore the effects of hydroxytyrosol on the piglet gut microbiota by adjusting the dosages, considering species specificity, studying piglets at different growth stages, and controlling experimental conditions.

The gut microbiota transforms PBA into SBA through various biotransformation processes, and BAs in turn affect the composition and abundance of the gut microbiota, establishing a bi-directional relationship known as the gut microbiota–bile acid axis [54]. This interplay illustrates the dynamic interaction and mutual influence between host bile acid and the gut microbiota. In this study, we observed that hydroxytyrosol had a limited effect on restoring the imbalance of the microbiota caused by oxidative stress. Nevertheless, a notable finding in the study results was a significant alteration in the composition of BAs. Specifically, oxidative stress led to lower levels of several key BAs, such as HCA, HDCA, and TUDCA. This result indicated that oxidative stress interferes with BAs homeostasis. Notably, treatment with hydroxytyrosol partially reversed these changes, reminiscent of the effects of other polyphenolic compounds, such as caffeic acid, in lipopolysaccharide (LPS)-induced intestinal injury models [55]. This phenomenon is intriguing and presents challenges for interpretation, which may be attributed to several mechanisms: Firstly, direct action on BA metabolism: Hydroxytyrosol may directly affect certain key enzymes or transporters involved in the synthesis, conversion, or excretion of BAs, thereby improving their metabolism. For instance, resveratrol has been found to modulate BA metabolism by altering the activity of microbial enzymes [12,56]. Secondly, indirect impact on microbial metabolism: Although hydroxytyrosol did not significantly alter the changes in the gut microbiota induced by oxidative stress, it may have improved the intestinal environment, thereby influencing the metabolic activities of the microbiota and their potential implications for BA metabolism. Thirdly, improvement of intestinal cell function: Hydroxytyrosol may have reduced oxidative stress-induced damage to intestinal cells, thereby maintaining their functional integrity and influencing the synthesis and secretion of BAs. Lastly, maintenance of intestinal barrier function: Hydroxytyrosol may have contributed to the maintenance of intestinal barrier function, reducing the damage from oxidative stress to intestinal cells and thereby preserving normal BA metabolism. Previous studies have suggested that impairment of the intestinal barrier function and exacerbated inflammation may affect the absorption and metabolism of BAs [57,58]. The findings presented here open avenues for further investigation into the complex interplay between oxidative stress, gut microbiota, and BAs metabolism, and the potential therapeutic applications of hydroxytyrosol in maintaining intestinal health.

HCA and HDCA are hyocholic acid species that have shown the potential to improve glucose homeostasis in metabolic diseases [59]. Currently, there is limited research on the role of HCA in regulating intestinal oxidative damage. Our study found a positive correlation between the expression levels of HCA, Nrf2, and CAT, indicating its potential antioxidant function. Meanwhile, pretreatment with HDCA significantly reduced blood–brain barrier permeability and neuronal apoptosis. It also enhanced the activity of the antioxidant enzyme γ-glutamyl transferase, thereby mitigating oxidative stress injury and the release of inflammatory cytokines. As a result, it maintained the morphological integrity and function of the neurovascular unit [60]. Furthermore, recent studies have suggested that HDCA may act as an endogenous inhibitor of inflammatory signals [61]. These results suggest that HDCA may also have the potential to improve intestinal oxidative damage. TUDCA has been primarily studied for its antioxidant properties. Many studies have confirmed that TUDCA can regulate the activity of antioxidant enzymes to decrease oxidative stress [62,63,64]. Further support for this finding was provided by the positive correlation observed between TUDCA and the expression levels of Nrf2 and CAT. Previous research has also found that BAs can activate the Nrf2 signaling pathway [65]. In addition, our study also found higher levels of LCA after hydroxytyrosol treatment. The exact reasons for this phenomenon are not yet fully understood. It is possible that hydroxytyrosol, commonly used in the treatment of high cholesterol and cardiovascular diseases, may lower blood cholesterol levels by regulating cholesterol metabolism pathways, which may include reducing the synthesis or increasing the excretion of LCA, thereby leading to a lower level of LCA [66]. It is thus possible that hydroxytyrosol alleviates intestinal oxidative damage by regulating BA metabolism. The results of this study provide important basic data for further exploration of the effects of hydroxytyrosol on BA metabolism and the response to oxidative stress. These findings not only enhance our understanding of BA metabolism and its changes under oxidative stress but also provide a new perspective for the development of potential antioxidant therapies.

This study has made preliminary progress in exploring the positive impact of hydroxytyrosol on intestinal oxidative damage, but several apparent limitations need to be overcome in future research. First, although the improvement of BA metabolism by hydroxytyrosol has been observed, the specific regulatory mechanism is still not fully understood. Particularly, this study did not find significant changes in the gut microbiota, which is generally considered to play a key role in BA metabolism. Therefore, future research can use advanced techniques such as metagenomics to analyze changes in BA synthesis-related enzyme activity and potential molecular pathways to elucidate the regulatory mechanism of hydroxytyrosol. Further analysis of molecular changes related to BA synthesis pathways in the liver is also warranted. Secondly, this study did not delve into the effects of hydroxytyrosol on intestinal oxidative damage at the protein or tissue level. Future research can utilize molecular biology methods such as Western blot and immunohistochemistry to analyze the effects of hydroxytyrosol more intricately on markers of intestinal injury, providing a more comprehensive understanding of the physiological mechanisms. Thirdly, the sample of this study was limited to piglets and did not provide clinical data support. To validate the universality of these findings, future research should include clinical trials to evaluate the effects of hydroxytyrosol in a broader population. Lastly, the combined application of hydroxytyrosol with other antioxidants may enhance therapeutic efficacy. Further research can explore the synergistic effects of different drug combinations on BA metabolism and intestinal health to optimize treatment strategies. Overall, while the current research provides valuable clues for future research directions, a series of meticulously designed experiments are still needed to enrich and validate these preliminary findings, to provide a solid scientific basis for the clinical application of hydroxytyrosol.

**Figure 5:**
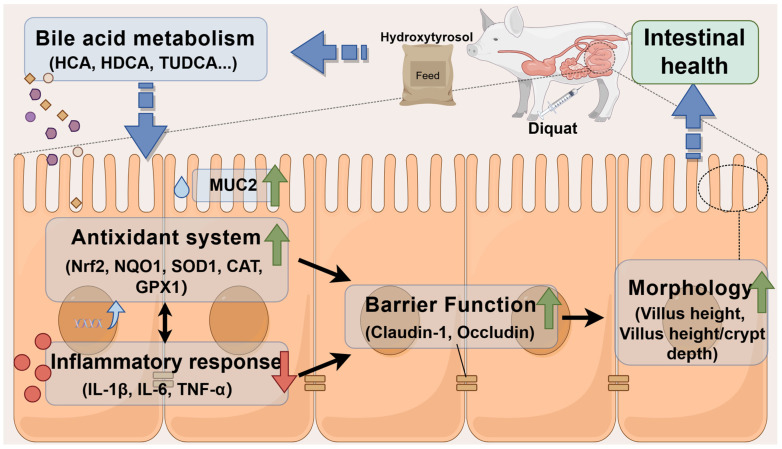
The potential mechanism of hydroxytyrosol in alleviating intestinal oxidative stress and improving intestinal health. Green arrows indicate an increase, while red arrows indicate a decrease.

In summary, our study found that oxidative stress can lead to intestinal damage, and hydroxytyrosol can alleviate these adverse effects. This is because hydroxytyrosol regulates BA metabolism, which in turn modulates the expression of antioxidant genes, reduces inflammatory cytokines, promotes intestinal barrier integrity, and ultimately maintains intestinal health and homeostasis (Figure 5). These findings provide important insights for the development of therapeutic strategies targeting intestinal oxidative stress, emphasizing the potential of hydroxytyrosol as a potential drug in alleviating oxidative damage and treating intestinal diseases. Additionally, it is recommended to consume foods rich in hydroxytyrosol to prevent and improve intestinal oxidative damage in infants and young animals.

## 4. Materials and Methods

### 4.1. Experimental Design

A total of 24 healthy weaned piglets (21 days old; 7.66 ± 0.85 kg; Duroc × Landrace × Yorkshire; male) were randomly assigned to 4 treatment groups: control group (CON), diquat group (DQ), hydroxytyrosol group (HT), and HT+DQ group (HTD). The CON and DQ groups were fed the basal diet, whereas the HT and HTD groups were fed the basal diet supplemented with 500 mg/kg HT (≥99%, Hangzhou Viablife Biotech Co., Ltd., Hangzhou, China). The basal diet met the NRC (2012) nutrient requirements for piglets (Supplementary Table S1). The amount of daily feed was estimated at 5% of body weight. All piglets were housed in a clean and comfortable environment with the availability of food and water *ad libitum*. The experiment lasted for 28 days. On the 21st day, all piglets in the DQ and HTD groups were intraperitoneally injected with DQ (8 mg/kg body weight; Dr. Ehrenstorfer, Augsburg, Germany), while all piglets in the CON and HT groups were intraperitoneally injected with normal saline in an equal volume. Subsequently, the piglets continued to be managed and fed as per the original protocol until the end of the experiment. The selection of sample size is based on the review of literature, previous research opinions, and careful consideration of practical and ethical factors [39,67]. The selection of HT concentration is based on our previous pre-experiment, while the selection of DQ concentration is referenced from the study by Xu et al. (2008) [68].

### 4.2. Sample Collection

On the 28th day, all piglets were anesthetized with intravenous injections of pentobarbital sodium and euthanized by exsanguination through the neck for a humane procedure. After opening the abdominal cavity of piglets, the intestine was quickly separated, and a 2 cm ileum tissue section was taken from about 15 cm away from the ileocecal part and fixed in 4% paraformaldehyde solution. Additionally, a segment of ileum was collected at the same location, ileal digesta was collected, washed with pre-chilled normal saline solution, and ileal mucosa was gently scraped with a surgical blade, placed in 2 mL sterile cryopreservation tubes, and flash-frozen in liquid nitrogen before storage at −80 °C for subsequent analysis.

### 4.3. Histological Analysis

After 24 h of fixation, the ileal tissue was embedded in paraffin, sectioned at a thickness of 5 μm, and stained with a hematoxylin eosin (HE) staining kit (Solarbio, Beijing, China). Subsequently, 15 typical visual fields displaying intact villi and crypt and a straight orientation, were selected for histological examination under a Leica DM2000 light microscope. For histological examination, two experienced authors jointly observed the ileal characteristics and structures and reached conclusions based on consensus. Villus height and crypt depth were meticulously measured, and villus height/crypt depth was calculated by dividing the villus height by crypt depth.

### 4.4. Quantitative Real-Time PCR (qRT-PCR)

RNA extraction and qRT-PCR were carried out according to the previously described methods [55]. Briefly, RNA was extracted from the ileum by RNeasy Kit (Genebetter, Beijing, China), then quantified by Nanodrop 2000, and reverse transcription into cDNA by Reverse Transcription Kit (Takara, Kusatsu, Japan). Finally, qRT-PCR was performed using a TB green kit (Takara, Kusatsu, Japan). Tight junction protein (ZO-1, Occludin and Claudin-1), cytokines (IL-1β, IL-6, IL-10 and TNF-α), Nrf2 and its downstream genes (Nrf2, Keap1, HO-1, NQO1, SOD1, CAT, GPX1, and GPX2), and MUC2 were analyzed. β-actin and GAPDH were used as internal reference genes, and the gene expression was quantitatively calculated by the 2^−△△CT^ method. All gene sequences were queried using NCBI databases, and primers were designed using the Primer-BLAST tool. The primers were synthesized by Shanghai Sangon Biotech (Shanghai, China). Furthermore, all primers have been evaluated for their specificity and amplification efficiency (90–105%). The specific primers used are described in Table 1.

### 4.5. Microbial 16S rRNA Analysis

Microbial DNA extraction, amplification of the V3-V4 variable region of the 16S rRNA gene, and sequencing were conducted by OE Biotech Company (Shanghai, China). Briefly, DNA was first extracted from ileal digesta. Then, universal primers 343F (5′-TACGGRAGGCAGCAG-3′) and 798R (5′-AGGGTATCTAATCCT-3′) were used to amplify the target region. The amplicon quality was visualized using agarose gel electrophoresis. Sequencing was performed on an Illumina NovaSeq 6000 with 250 bp paired-end reads (Illumina Inc., San Diego, CA, USA; OE Biotech Company; Shanghai, China). Gut microbiota was analyzed using OECloud tools at https://cloud.oebiotech.cn (accessed on 21 March 2024) to obtain the alpha diversity (Chao1, Goods coverage, observed species, ACE, Shannon, and Simpson) and beta diversity and composition of microorganisms. Linear discriminant analysis effect size (LDA Effect Size, LEfSe) was used to identify the maker bacteria of the individual group, and the threshold of the LDA score was 3.0.

### 4.6. Quantification of SCFAs and BAs

The detection and analysis of SCFAs and BAs in ileal digesta refer to our previous research [69,70]. SCFAs were detected as follows: Briefly, about 1 g of ileal digesta was dissolved in distilled water and shaken for 30 min. The mixture was then incubated at 4 °C overnight. The supernatant was obtained by centrifugation at 12,000× *g* for 10 min. Then, the supernatant was treated with metaphosphoric acid (25%, *w*/*v*) at a 1:9 ratio. Finally, after vortex and centrifugation, the supernatant was filtered through a 0.45 µm filter and SCFAs were quantified by Agilent 7890N GC, with a sample injection volume of 2 μL. The analysis was performed on a DB-FFAP column (15 m × 0.32 mm × 0.25 μm) with the following specific parameters: the column temperature was initially set at 100 °C, then increased at a rate of 2 °C/min to 120 °C and held constant for 10 min. The injection port and detector temperatures were maintained at 250 °C and 280 °C, respectively, with a constant pressure of 21.8 kPa and a split ratio of 1:50. Qualitative analysis was conducted using the retention times of SCFAs standards, and concentrations were calculated based on peak areas.

BAs were detected as follows: Briefly, a quantity of 50 mg of lyophilized digesta was suspended in a solution consisting of 500 μL of 50 mM sodium acetate buffer (pH 5.6) and 1.4 mL of methanol. Then, the suspension was incubated on an orbital shaker at 150× *g* for 1 h at 45 °C. After centrifugation at 20,000× *g* for 15 min, the supernatant was diluted 4-fold with sodium acetate buffer and filtered through a Bond Elute C18 cartridge (Agilent, Waldbronn, Germany). The cartridge was washed with 25% ethanol, and then BAs were eluted with 5 mL methanol. Subsequently, the eluate was dried using nitrogen gas and redissolved in 1 mL of methanol. The solution was filtered through a 0.45 µm filter and BAs were quantified by LC-MS/MS. The 10 µL supernatant was injected into a ZORBAX Eclipse plus C18 column (95 Å, 1.8 µm, 2.1 × 100 mm) to separate BAs. The mobile phase consisted of 5% acetonitrile and 0.1% formic acid (mobile phase A) and 95% acetonitrile and 0.1% formic acid (mobile phase B). The elution gradient for BA was gradually changed as follows: mobile phase A: B (9:1, *v*/*v*) from 0 to 1 min, mobile phase A: B (7:3, *v*/*v*) from 1 to 1.5 min, mobile phase A: B (2:3, *v*/*v*) from 1.5 to 5.5 min, mobile phase A: B (7:3, *v*/*v*) from 1.5 to 5.5 min, and mobile phase A: B (9:1, *v*/*v*) from 5.5 to 7 min. The spray voltage and ion source temperature were set at 2.91 kV and 500 °C, respectively, with a gas flow rate of 550 L/h. Quantification of each BA was based on a standard curve generated from the series gradient dilutions of existing standard samples.

### 4.7. Statistical Analysis

Firstly, we conducted Shapiro–Wilk and Levene’s tests on the data to determine if it met the requirements of normal distribution and homogeneity of variance. If the data met the criteria for normal distribution and homogeneity of variance, we proceeded with one-way analysis of variance (ANOVA) coupled with the Tukey test for further analysis. For data that did not meet these criteria, we employed the Kruskal–Wallis test for statistical analysis. Data Integration Analysis for Biomarker discovery using a Latent component method for Omics (DIABLO) was performed for correlation analysis using the package “mixOmics” in the R program. All statistical significance was set at *p* < 0.05.

## Figures and Tables

**Table 1:** Primers for qRT-PCR analysis.

Genes	Accession No.	Primer Sequences
ZO-1	XM_003480423.4	F: GGGGTAGGGGTCCTTCCTAT R: CATTTGCTTGGCAGTCAGGTT
Occludin	NM_001163647.2	F: CAGGTGCACCCTCCAGATTG R: TATGTCGTTGCTGGGTGCAT
Claudin-1	NM_001243483.1	F: TTTCCTCAATACAGGAGGGAAGC R: CCCTCTCCCCACATTCGAG
MUC2	XM_021082584.1	F: CGCATGGATGGCTGTTTCTG R: ATTGCTCGCAGTTGTTGGTG
IL-1β	NM_214055.1	F: CCAGCCAGTCTTCATTGTT R: CATCTCTTTGGGGCCAT
IL-6	NM_001252429.1	F: TCCAATCTGGGTTCAATCA R: TCTTTCCCTTTTGCCTCA
IL-10	NM_214041.1	F: CCGACTCAACGAAGAAGG R: CTGGTTGGGAAGTGGATG
TNF-α	NM_214022.1	F: CCGACAGATGGGCTGTA R: TCTTGATGGCAGAGAGGAG
Nrf2	XM_013984303.2	F: TAAGGGTGCTCCTTTGCGAG R: ATCAAATCCATGTCCTTGGCG
Keap1	XM_021076667.1	F: CGCCTCATCGAGTTCGCTTACAC R: GCACGGACCACACTGTCAATCTG
HO-1	NM_001004027.1	F: GTTTGAGGAGGTGCAGGAG R: GGTTGTCACGGGAGTGG
NQO1	NM_001159613.1	F: CAGCATTGGGCACACTC R: GGCGCAAAGTACAGTGG
SOD1	NM_001190422.1	F: GCCAAAGGATCAAGAGAGG R: GAGAGGGCGATCACAGAA
CAT	NM_214301.2	F: GCTGAGTCCGAAGTCGTCTA R: GTCAGGATATCAGGTTTCTGCG
GPX1	NM_214201.1	F: AACGGTGCGGGACTACA R: TCGGACGTACTTGAGGCA
GPX2	NM_001115136.1	F: CAACACATTCCGAGGCA R: GAAGCCAAGAACCACCAG
β-actin	XM_001928093.1	F: GCGTAGCATTTGCTGCATGA R: GCGTGTGTGTAACTAGGGGT
GAPDH	NM_001206359.1	F: CGTGTCGGTTGTGGATCTGA R: TGACGAAGTGGTCGTTGAGG

## Data Availability

Raw reads for 16S rRNA gene sequencing were submitted to the NCBI Sequence Read Archive database (Accession Number: PRJNA960710).

## Supplementary Materials

The following supporting information can be downloaded at: https://www.mdpi.com/article/10.3390/ijms25115590/s1.
