# Supplementary material for: Hydroxytyrosol Alleviates Intestinal Oxidative Stress by Regulating Bile Acid Metabolism in a Piglet Model
Source: Int J Mol Sci. 2024 May 21;25(11):5590. doi: 10.3390/ijms25115590 (PMC11171822; doi:10.3390/ijms25115590)
Supplement: Supplementary file 1 [file ijms-25-05590-s001.zip › ijms-2976567-supplementary.pdf]

Table S1. The composition and nutrient level of basal diet (air-dry basis)

Item	%
**Ingredients**	
Suckling corn	28.98
Expanded corn	15.00
Broken rice	15.00
Expanded soybean	13.00
Dried whey	8.00
Soybean meal	4.50
Glucose	4.00
Fish meal	3.00
Hydrolyzed Wheat Protein	3.00
Soybean oil	1.50
Calcium Formate	0.80
Montmorillonite	0.50
Calcium phosphate monobasic	0.50
Lysine	0.65
Thr	0.24
Met	0.16
Trp	0.07
Ethoxyquin	0.10
Premix[1]	1.00
**Nutrient levels[2]**	
Net energy, kcal/kg	2,620
Crude protein	17.39
Calcium	0.69
Phosphorus	0.54
Lysine	1.23
Methionine	0.57
Threonine	0.90
Cystine	0.26

[1] The premix provides per kg of diet: VA: 15,000 IU; VD_3: 4,500 IU; VE: 72.5 mg; VK_3: 4.5 mg; VB_1: 4.32 mg; VB_2: 12 mg; VB_6: 4.86 mg; VB_{12}: 30 μg; Biotin: 480 μg; Folic acid: 1.764 mg; Calcium Pantothenate: 19.32 mg; Nicotinamide: 41.58 mg; Cu: 110 mg; Fe: 165 mg; Zn: 80 mg; Mn: 60 mg; I: 0.8 mg; Co: 0.6 mg; Se: 0.3 mg.

[2] Net energy and Crude protein are calculated values, while the other nutrient levels are measured values.

Table S2. Standard information of short chain fatty acids and bile acids

Item	Molecular formula	Cat
**Short chain fatty acids**		
Acetate	CH3CO2H	71251
Propionate	CH3CH2COOH	94425
Isobutyrate	(CH3)2CHCO2H	I1754
Butyrate	CH3CH2CH2COOH	19215
Isovalerate	(CH3)2CHCH2COOH	78651
Valerate	C5H10O2	30191428
**Bile acids**		
Hyocholic acid (HCA)	C24H40O5	C1850-000
Hyodeoxycholic acid (HDCA)	C24H40O4	H3878
Chenodeoxycholic acid (CDCA)	C24H40O4	C9377
Tauro-chenodeoxycholic acid (TCDCA)	C26H44NNaO6S	T6260
Tauro-ursodeoxycholic acid (TUDCA)	C26H44NO6SNa	T0266
Cholic acid (CA)	C24H40O5	C1129
Glycocholic acid (GCA)	C26H43NO6·xH2O	G2878
Lithocholic acid (LCA)	C24H40O3	L6250
7-Ketolithocholic acid (7-KLCA)	C24H38O4	700238P
12-Ketolithocholic acid (12-KLCA)	C24H38O4	700239P
α-Muricholic acid (α-MCA)	C24H35D5O5	908223
β-Muricholic acid (β-MCA)	C24H35D5O5	908231
Tauro-ω-muricholic acid (Tω-MCA)	C26H44NNaO7S	700245P
